# Noncoding RNAs: Novel Targets for Opioid Tolerance

**DOI:** 10.2174/1570159X21666221129122932

**Published:** 2023-04-12

**Authors:** Meiling Deng, Wangyuan Zou

**Affiliations:** 1 Department of Anesthesiology, Xiangya Hospital, Central South University, Changsha, 410008, Hunan, China;; 2 Department of Anesthesiology, The First Affiliated Hospital of Chongqing Medical University, Chongqing, China;; 3 National Clinical Research Center for Geriatric Disorders, Xiangya Hospital, Central South University, Changsha, 410008, Hunan, China

**Keywords:** Noncoding RNA, chronic pain, opioid tolerance, microRNA, long noncoding RNA, circRNA

## Abstract

As a global health problem, chronic pain is one of the leading causes of disability, and it imposes a huge economic and public health burden on families and society. Opioids represent the cornerstone of analgesic drugs. However, opioid tolerance caused by long-term application of opioids is a major factor leading to drug withdrawal, serious side effects caused by dose increases, and even the death of patients, placing an increasing burden on individuals, medicine, and society. Despite efforts to develop methods to prevent and treat opioid tolerance, no effective treatment has yet been found. Therefore, understanding the mechanism underlying opioid tolerance is crucial for finding new prevention and treatment strategies. Noncoding RNAs (ncRNAs) are important parts of mammalian gene transcriptomes, and there are thousands of unique noncoding RNA sequences in cells. With the rapid development of high-throughput genome technology, research on ncRNAs has become a hot topic in biomedical research. In recent years, studies have shown that ncRNAs mediate physiological and pathological processes, including chromatin remodeling, transcription, posttranscriptional modification and signal transduction, which are key regulators of physiological processes in developmental and disease environments and have become biomarkers and potential therapeutic targets for various diseases. An increasing number of studies have found that ncRNAs are closely related to the development of opioid tolerance. In this review, we have summarized the evidence that ncRNAs play an important role in opioid tolerance and that ncRNAs may be novel targets for opioid tolerance.

## 1 INTRODUCTION

Chronic pain usually lasts more than 3 months [1] and has become a worldwide health problem and the main cause of disability [2]. According to epidemiological statistics, the incidence of chronic pain is as high as 20% to 50% worldwide. In the United States, chronic pain affects one in three Americans and costs the American economy $635 billion annually [3]. However, the incidence of chronic pain continues to rise as the global population ages, diabetes rates rise, and survival rates improve for cancer patients undergoing chemotherapy [4].

Chronic pain causes severe pain, sleep disturbance, anxiety and depression [5], which not only seriously affects the quality of patients’ lives but also places heavy economic and public health burdens on families and society [6, 7]. Opioids provide excellent pain relief and have been widely used for many years to treat a variety of moderate to severe pain conditions. The urgency of the medication needs of pain patients, the effectiveness of opioids for pain relief, and the limited treatment alternatives for chronic pain have led to excessive dependence on opioids, and the associated problems of tolerance are alarming [8].

Opioid tolerance refers to the decrease in the body’s response to opioids after repeated administration and the need to increase the dose to achieve the same analgesic effect. However, increased doses of opioids are associated with an increased risk of addiction, respiratory depression and even death [9]. Therefore, opioid tolerance is the key to the failure of opioid analgesic therapy. The mechanisms of opioid tolerance involve multiple organs and systems, such as the brain, spinal cord, and dorsal root ganglia (DRG). In addition, changes in neurotransmitters and other molecules, receptors, channels and signaling pathways are also crucial to its development [10-12]. Although the specific mechanism of opioid tolerance has been explored, opioid tolerance is still a global public health problem, and further research is needed to find new effective targets to overcome it.

The understanding of the RNA world inside mammalian cells has been expanding for decades, and each discovery opens up a new and surprising landscape for biological regulation and function [13]. With the rapid development of high-throughput genome sequencing and microarray technologies, it has been found that the protein-coding genome covers only 2% of the entire genome, meaning that only a small fraction of RNA is translated into functional proteins [14, 15]. Noncoding RNAs (ncRNAs) are transcripts that do not normally encode proteins. ncRNAs include microRNAs (miRNAs), long noncoding RNAs (lncRNAs), circular RNAs (circRNAs), intronic RNAs and enhancer RNAs, and increasing evidence indicates that ncRNAs play an important role in the regulation of gene expression [16]. For example, ncRNAs can mediate posttranscriptional gene silencing, leading to mRNA degradation and inhibiting protein translation [17]; ncRNAs reconstruct the chromatin structure by guiding heterochromatin formation [18], and ncRNAs can also enhance or inhibit gene expression by regulating genes through *cis* or *trans* action [19]. These ncRNAs may be involved in the pathological process of various diseases, such as tumors [20], Alzheimer’s diseases [21], spinal cord injury [22], cardiovascular diseases [23] and chronic pain [24], making them a new class of drug therapy targets. Recently, several studies have focused on the function of ncRNAs in opioid tolerance, and some studies have shown that ncRNAs are participants in the mechanism of opioid tolerance [25-28], which opens up a new strategy for the prevention and treatment of opioid tolerance.

In this review, we focus on the frontier findings of ncRNAs and their expression changes in the brain, spinal cord and dorsal root ganglion (DRG) in preclinical rodent models of opioid tolerance. We also provide evidence of how opioids induce the dysregulation of ncRNAs in relevant regions and how dysregulated ncRNAs contribute to the pathogenesis of opioid tolerance. This review provides up-to-date knowledge about the role of ncRNAs in opioid tolerance and their specific mechanisms.

## 2 NONCODING RNAs AND OPIOID TOLERANCE

Opioids, such as morphine, have been found to be the most effective painkillers for pain since they were isolated from poppies in the early 19th century. However, repeated or long-term application of opioids leads to problems, such as tolerance and hyperalgesia, while side effects, such as respiratory depression and pruritus caused by increased doses, seriously reduce their effectiveness and safety [29]. In some cases of tolerance, even increasing opioid doses to the highest non-toxic dose did not provide the desired pain relief [30]. The emergence and development of opioid tolerance not only significantly reduces its analgesic efficacy but also requires increasing the opioid dose to combat the tolerance, increasing the risk of opioid addiction, which is a major medical and public health problem [31]. Many efforts have been made to elucidate the mechanisms of opioid tolerance [32, 33], and there is increasing evidence for the role of transcription and epigenetic regulation in opioid tolerance, such as the activation and inhibition of transcription factors [34], chromatin and DNA structure modification [35], and ncRNAs, including miRNAs, lncRNAs, and circRNAs [36].

## 3 miRNA AND OPIOID TOLERANCE

### 3.1 Biological Characteristics and Functions of miRNAs

miRNAs are noncoding single-stranded RNA molecules with a length of approximately 21-22 nucleotides [37]. The genes transcribed in the nucleus are catalyzed by RNA polymerase II to generate pri-miRNAs that are processed into precursor miRNAs (pre-miRNAs) [38]. Subsequently, nuclear transporters transport the pre-miRNAs from the nucleus to the cytoplasm [39]. The RNase III endonuclease Dicer and helper proteins promote the production of incompletely matching double-stranded miRNA‒miRNA; one is named the guide strand, and the other is named the passenger strand. The guide strand is incorporated into the miRNA-induced silencing complex (miRISC) under the action of miRNA guide protein Argonaute, and the passenger strand is degraded [40, 41]. miRNAs regulate gene expression mainly by binding to the 3’-untranslated region (3’-UTR) of target mRNAs. In most animals, miRNAs bind to the incomplete bases of the 3’-UTR sequence and inhibit protein synthesis by inhibiting translation or promoting deadenylation and degradation of the target mRNAs [42]. miRNAs are considered to be important regulatory factors involved in various cellular activities and diseases.

### 3.2 miRNAs and Opioid Tolerance

The nervous system is a rich source of miRNAs, which have multiple functions in basic neurobiological processes, such as neuronal development, plasticity, metabolism and apoptosis. miRNAs regulate gene expression at the posttranscriptional level in the nervous system. Increasing numbers of studies have shown that opioid administration leads to changes in the expression levels of a variety of miRNAs in the brain, spinal cord and DRG, thereby regulating the expression of downstream target proteins and participating in opioid tolerance [43].

#### 3.2.1 miRNAs Expressed in the Brain

Opioid receptors belong to the G protein-coupled receptor (GPCR) family [44]. Morphine acts on the mu-opioid receptor (MOR) to activate G protein conjugation to opioid receptors and induces dissociation of Gα and Gβγ subunits [45]. Then, the voltage-gated calcium channel is inhibited, the inward rectifying potassium channel is activated, the downstream adenylate cyclase (AC) is inhibited, and the cyclic adenosine monophosphate (cAMP) level is reduced. This inhibits the activation of the protein kinase A (PKA) pathway, which leads to decreased neurotransmitter release, resulting in analgesic effects [46]. After opioids bind to the receptors, G protein-coupled receptor kinases (GRKs) stimulate the phosphorylation of the receptors, leading to the continuous binding of β-arrestin-2 to MORs and the desensitization, internalization and degradation of receptors, resulting in a reduced analgesic effect, increased pain intensity, tolerance and opioid-induced hyperalgesia [47].

Opioid tolerance is a chronic adaptation induced by long-term or repeated administration of opioids, and it is mediated by complex changes in MORs at the molecular, synaptic, cellular and circuit levels [48]. Studies have reported that chronic opioids can lead to a significant decrease in MOR density and downregulation of MOR at high-affinity loci in the brain [49]. Some studies have reported that chronic opioid application does not significantly affect the level of MOR in the brain [50]. Other studies have shown that the expression of MOR in the brain was upregulated after long-term administration of opioids [51]. In general, the development of opioid tolerance is closely related to the number and function of MORs.

The let-7 family includes the first confirmed human miRNA, and its members are highly conserved in sequence and function. The let-7 family has been found to have ubiquitous sequences that partially complement the 3’-UTR of MOR mRNA and function as key regulators of MOR in opioid tolerance [31]. He *et al.* [49] studied the function of the let-7 family in cell and animal models of morphine tolerance and found that chronic morphine treatment resulted in increased expression of let-7 and decreased expression of MOR *in vitro*. In addition, the expression of let-7 in the brains of mice gradually increased over time after morphine treatment, which was time-correlated with the development of morphine tolerance. Let-7 was upregulated in mouse cortical cells that expressed MOR but not in cells not expressing MOR. The application of a let-7 inhibitor could decrease the levels of let-7 in the brain and partially attenuate morphine tolerance. Lu *et al.* [52] found that chronic morphine treatment significantly upregulated the expression of miR-103 and miR-107 and decreased the expression of polyribosomal-associated MOR-1A in both Be(2)C cells and the striatum of morphine-tolerant mice. The interaction between miR-103/107 and MOR-1A may contribute to the development of morphine tolerance. It was also found that chronic morphine treatment significantly increased the expression of miR-378-3p in Be(2)C cells and the brainstem of morphine-tolerant mice, resulting in decreased expression of MOR-1B3 and MOR-1B4 [53]. These studies provide a new perspective for understanding the regulatory role of miRNAs in morphine tolerance. In conclusion, the chronic application of opioids may upregulate the expression of some miRNAs in the brain, and these miRNAs complement and combine with part of the 3’-UTR of MOR mRNA, inhibiting the translation and biosynthesis of MOR and promoting the formation of opioid tolerance.

In addition to the upregulation of miRNAs promoting opioid tolerance, the downregulation of miRNAs also contributes to opioid tolerance. For example, Tapocik *et al.* [54] found that repeated administration of morphine decreased the expression of miR-27a in the prefrontal cortex of mice, and the downregulation of miR-27a led to a reduction in Serpin1 expression, leading to morphine tolerance. They found that miR-27a upregulates the expression of Serpin1 mRNA and protein, contrary to the fact that in most cases, miRNAs bind to target mRNAs to inhibit their translation and reduce protein levels. Studies on miRNAs activating translation have also been reported; these miRNAs can bind to adjacent/overlapping *cis*-acting sites rich in Au motifs in the 3’-UTR of the target mRNAs, thus improving the translation efficiency of the target mRNAs; however, the mechanism remains to be further elucidated [55-57].

#### 3.2.2 miRNAs Expressed in the Spinal Cord

Researchers have found that some abnormally expressed miRNAs in the spinal cord are involved in opioid tolerance. In addition to abundant secondary sensory neurons, the dorsal horn also expresses high concentrations of MORs [58]. Nevertheless, MORs are not the only target of miRNAs, and miRNAs also participate in opioid tolerance by regulating receptors, ion channels and protein kinases.

Toll-like receptor 4 (TLR4) is an important immune receptor in the central immune system; it participates in the recognition process of pathogen-related molecular patterns and resists the invasion of pathogens, playing a key role in the innate immune response, and this eventually leads to the production of inflammatory cytokines, type I interferon, chemokines and antimicrobial peptides through a series of signal transduction [59]. After being activated by morphine, TLR4 activates microglia and then increases the synthesis and release of cytokines, thus increasing the excitability of pain-sensing cells and reducing the analgesic effect of morphine. Inhibition of TLR4 signaling could improve the analgesic effect of morphine and relieve morphine tolerance [60]. miR-146a not only has an immunomodulatory function but also participates in the development of hematopoiesis, tumors and other physiological and pathological processes [61-63]. Recently, it has been found that miR-146a participates in opioid tolerance by regulating TLR4. Wang *et al.* [64] found the expression of miR-146a to be significantly decreased in the spinal cord of rats repeatedly treated with morphine, while the expression of TLR4 and its downstream molecule tumor necrosis factor receptor-associated factor-6 (TRAF6) was upregulated with an increased dose of morphine. Overexpression of miR-146a enhanced the analgesic effect of morphine and attenuated the formation of morphine tolerance by inhibiting the increase in TLR4 and TRAF6. Similarly, in the dezocine tolerance model of rats, miR-124-3p was significantly downregulated in the spinal cord, and overexpression of miR-124-3p alleviated dezocine tolerance by inhibiting TRAF6 [65].

NOD-like receptor protein 3 (NLRP3) is part of an inflammatory body that can be activated by cathepsin [66]. Researchers have shown that morphine stimulates TLR4 receptors in microglia and can induce upregulation of the NLRP3 inflammasome, thereby promoting morphine tolerance [67]. NLRP3 is one of the targets of miR-223, and miR-223 participates in the development of the inflammatory response by negatively regulating NLRP3 expression [68, 69]. It has been reported that overexpression of miR-223 in the spinal cord could attenuate morphine tolerance in rats by inhibiting the expression and activity of NLRP3 [70].

miR-365 participates in the differentiation, proliferation and apoptosis of cancer cells and endothelial cells [71]; it is also expressed in the central nervous system and is involved in neurological dysfunction [72]. Wang *et al.* [73] found miR-365 to be significantly downregulated in the spinal cord of morphine-tolerant rats. Overexpression of miR-365 could prevent and partly reverse morphine tolerance by inhibiting β-arrestin-2. In addition, Wu *et al.* [74] found that miR-365 in the spinal cord could decrease the expression of IL-1β, TNF-α and IL-18 by targeting β-arrestin-2 and inhibit the activation of astrocytes and the ERK/CREB signaling pathway, thus alleviating the development of morphine tolerance. Calmodulin-dependent protein kinase II γ (CaMKII γ), a component of the CaMKII family, is a multifunctional protein kinase highly expressed in the central nervous system. CaMKII dysfunction has been shown to play an important role in drug addiction, depression, epilepsy, and various neurodevelopmental disorders [75]. CaMKII has been reported to modulate opioid tolerance through its effects on learning and memory, and the application of CaMKII inhibitors can reverse opioid tolerance [76]. Moreover, studies have shown that miR-219 regulates NMDA receptor-mediated neurobehavioral dysfunction and neuropathic pain by targeting CaMKII γ [77]. Wang *et al.* [78] found that chronic morphine treatment downregulated the expression of miR-219-5p and upregulated the expression of CaMKII γ in the spinal cord of rats, thereby increasing the expression and activity of NMDA receptors and leading to morphine tolerance. Overexpression of miR-219-5p could inhibit the translation of CaMKII γ and alleviate morphine tolerance.

As a classic negative immunoregulatory protein, A20 inhibits NF-κB signaling. ABIN1, an adaptor of A20, not only inhibits the phosphorylation and internalization of MOR but also inhibits the activation of NF-κB signaling after chronic morphine administration [79]. Huang *et al.* [80] found that long-term administration of morphine could significantly increase the expression of miR-873a-5p in the spinal cord of mice, which was negatively correlated with the decreased expression of A20. Downregulation of miR-873a-5p and overexpression of A20 partially reversed the development of morphine tolerance. Downregulation of miR-873a-5p in the spinal cord may represent a potential strategy to alleviate opioid tolerance.

CXC chemokine receptor 4 (CXCR4) is a widely expressed G-protein coupled receptor (GPCR) associated with human diseases, such as HIV. CXCR4 is mainly expressed in the membrane of neurons in the spinal dorsal horn, which activates neurons and promotes hyperalgesia by activating downstream intracellular signaling pathways [81]. Studies have shown that blocking the CXCR4 signaling pathway could alleviate inflammation and delay the development of morphine tolerance [82]. In a morphine tolerance model of bone cancer pain in rats, miR-338 in the spinal cord attenuated the formation of morphine tolerance by targeting CXCR4 [83]. These results suggest that miRNAs in the spinal cord may be targets for the prevention and treatment of opioid tolerance.

#### 3.2.3 miRNAs Expressed in the Dorsal Root Ganglion (DRG)

Pain signals are initially detected by nociceptors at the peripheral nerve fiber endings of primary sensory neurons located in the DRG and then transmitted to the central nervous system, including the spinal dorsal horn and pain-related brain regions, through the primary afferent nerve [84]. The expression and functional changes of neurochemical signals in DRG neurons are responsible for the formation of opioid tolerance [85]. There is evidence that dysregulated miRNAs in the DRG are also involved in opioid tolerance.

The JAK kinase/signal transducer and activator of transcription 3 (STAT3) signaling pathway can be activated after peripheral nerve injury and produce a neuroinflammatory response. Rapid and persistent activation of the JAK2/ STAT3 pathway causes neuropathic pain [86], and studies have reported that miR-375 can inhibit the JAK2/STAT3 pathway and reduce cell migration and proliferation [87]. Li *et al.* [88] found that after chronic morphine treatment, miR-375 was downregulated and JAK2 was upregulated in the DRG, and overexpression of miR-375 could significantly inhibit morphine tolerance. These results indicate that miRNAs in the DRG may be specific targets for the prevention and treatment of opioid tolerance.

All of these previous functional studies of differentially expressed miRNAs in the brain, spinal cord, and DRG during opioid tolerance (Table **1**) strongly support the important role of miRNAs in the biology of opioid tolerance. The ability of these selected miRNAs to target a variety of mRNAs that change during the development of opioid tolerance makes these miRNAs interesting candidates for therapeutic agents in the form of miRNA mimics or anti-miRNAs.

## 4 LncRNA AND OPIOID TOLERANCE

### 4.1 Biological Characteristics and Functions of lncRNAs

LncRNAs are a class of RNA transcripts with more than 200 nucleotides and no obvious protein-coding ability. Usually, they are transcribed from genes under the catalysis of RNA polymerase II, with a 5’-terminal methylguanosine cap structure and a 3’-terminal polyadenylate tail [89]. LncRNAs can be generated by chromosome rearrangement, reverse transcription during noncoding RNA replication, local tandem repeats and insertion of transposable factors [90, 91]. A major characteristic of lncRNAs is that they tend to fold into thermodynamically stable secondary and advanced structures, and in many cases, the secondary structure of the lncRNAs determines their function [92]. LncRNAs are widely distributed in different tissues, and some lncRNAs are preferentially expressed in specific tissues [93].

LncRNAs have different functional binding domains that can directly bind to DNA, RNA and proteins. Based on these interactions, they can be classified into the guide, decoy, scaffold and enhancer molecules involved in the posttranscriptional and posttranslational regulation of gene expression. LncRNAs may be expressed in the nucleus, cytoplasm or both, and different locations of lncRNAs play different functions. LncRNAs located in the nucleus can activate or inhibit transcription by introducing chromatin modifiers and various transcription regulators into target gene promoters in a *cis* or *trans* manner [94, 95]. They can also function as decoys for specific chromatin modifiers, isolating them from promoters of target genes [96]. LncRNAs can exert transcriptional regulation by competing with transcription factors for binding DNA and/or binding to their DNA-binding domains [97]. LncRNAs are involved in the regulation of alternative splicing of pre-mRNA in the nucleus, thus influencing the selection of dominant isoforms [98]. In the cytoplasm, lncRNAs are associated with the posttranscriptional regulation process that affects the stability of transcripts [99] or determines whether transcripts are translated into proteins; lncRNAs act as scaffolds to bring two or more proteins into a complex [100]. Finally, a common mechanism affecting mRNA abundance is to reduce miRNA-mediated degradation of target mRNAs by acting as so-called “sponges” for miRNAs through the competitive endogenous RNA (ceRNA) mechanism [101].

### 4.2 LncRNA and Opioid Tolerance

An increasing number of studies have shown that lncRNAs are new and promising therapeutic targets for a variety of diseases, but their role in opioid tolerance has not been clearly defined. Some studies applied microarray technology to screen some differentially expressed lncRNAs in opioid tolerance animal models and conducted bioinformatics analysis. Altered lncRNA expression in opioid tolerance may lead to gene dysregulation of neurotransmitter receptors and signaling molecules, which may underlie neuroadaptations associated with opioid tolerance.

#### 4.2.1 LncRNAs Expressed in the Brain

The effects of morphine on the spinal cord and brainstem neurons are mainly analgesic [102]. Long-term or repeated opioid exposure alters the expression levels of some genes in the spinal cord and brain regions, including the midbrain, striatum, hippocampus, and cortex [103, 104]. Previous studies have shown that long-term exposure to opioids alters the expression of ncRNAs, which may be partly responsible for the sustained changes in gene expression after morphine treatment [105]. In addition, neurobiological adaptations associated with opioid tolerance include not only changes in ascending and descending pain pathways but also regulation of the reward circuits at the level of gene expression and ncRNA regulation [106]. To explore the association between lncRNAs in the brain region and opioid tolerance, Ahmadi *et al.* [107] established morphine tolerance models by subcutaneous injection of morphine in rats and detected the expression of lncRNA H19, BC1, MIAT1 and MALAT1 in the midbrain, striatum, hypothalamus, prefrontal cortex and hippocampus. They found that the changes in lncRNAs were different in different brain regions: the expression of H19 was increased in the midbrain but decreased in the striatum, hypothalamus and prefrontal cortex; MIAT1 was decreased in the midbrain but increased in the striatum and hypothalamus; BC1 was decreased in the midbrain and increased in the hypothalamus; and MALAT1 was decreased in the midbrain, striatum and hypothalamus. Additional studies on the biological function of lncRNAs in the midbrain, striatum, prefrontal cortex, hypothalamus, and hippocampus in relation to morphine tolerance are warranted because opioid tolerance studies are shifting to cellular and molecular neural adaptations in the central nervous system. Identifying lncRNAs altered in the brain will help unravel the molecular mechanisms underlying opioid tolerance.

#### 4.2.2 LncRNAs Expressed in the Spinal Cord

In the early stage, we conducted microarray analysis of the spinal cord in morphine-tolerant rats and found 136 lncRNAs to be differentially expressed. CeRNA analysis indicated that these lncRNAs might bind to some miRNAs to regulate the expression of downstream molecules. For example, MRAK150340 could bind to miR-219b to regulate the expression of toll-interacting protein Tollip and other downstream molecules, and MRAK161211 could bind to miR-133 to regulate the function of Usp13 [105]. These results suggest that differentially expressed lncRNAs have potential biological functions worthy of further study.

Opioids are known to produce analgesic effects mainly through MORs [108]. The desensitization, phosphorylation, internalization, and downregulation of MORs are thought to be related to opioid tolerance [109]. In our research, we found lncRNA MRAK159688 and REST to be upregulated and MOR as downregulated in the spinal cord of morphine-tolerant rats, and knockdown of MRAK159688 could alleviate the formation and development of morphine tolerance by inhibiting the upregulation of REST and increasing the expression level of MOR [110] (Table **1**). These results indicate that the downregulation of MOR is an important factor in the formation of morphine tolerance and that the regulation of MRAK159688 may be a new strategy for the prevention and treatment of morphine tolerance.

In addition to animal experiments, Qiu *et al.* [111] screened and identified differentially expressed lncRNAs related to morphine tolerance by mining public databases through bioinformation technology. For example, the expression levels of XR_006440, XR_009493, AF196267, MRAK150340 and MRAK037188 were downregulated and those of MRAK046606, XR_005988, DQ266361, UC.167- and UC.468+ were upregulated, and these differentially expressed lncRNAs and pivotal genes could provide new research targets for the diagnosis and treatment of morphine tolerance.

Although studies on the role of lncRNAs in opioid tolerance have just started, the results of preliminary studies indicate a possible direction of exploration. In addition to MRAK159688 siRNA, which may be a new RNA-based treatment for opioid tolerance, additional potential targets will be gradually discovered in the future.

## 5 CircRNA AND OPIOID TOLERANCE

### 5.1 Biological Characteristics and Functions of circRNA

circRNAs are a unique class of single-stranded, closed-loop RNA molecules that reverse splice the 3’ and 5’ ends through exon or intron cyclization to form a complete covalently closed ring structure [112]. circRNAs are diverse, abundant and often evolutionarily conserved [113]. Most circRNAs are expressed by protein-coding genes and consist of one or more exons [114]. All basic types of products of alternative splicing of linear RNA can be found in circRNAs, and some circRNAs also contain exons not included in linear RNAs [115]. circRNAs are generally expressed at lower levels than their linear RNAs, but they are the primary transcription product for many genes [116]. Except for circRNAs containing introns, most circRNAs are exported to the cytoplasm after formation [117].

circRNAs play an important regulatory role in biological activities at multiple levels, including the transcriptional, posttranscriptional and epigenetic levels, specifically regulating transcription, splicing and interactions with chromatin. CircRNA sponges can adsorb miRNAs to regulate mRNA translation, and they can also act as scaffold molecules for proteins or directly combine with proteins to regulate their function [118, 119]. Numerous studies have revealed the unique expression characteristics and key biological roles of circRNAs in a variety of diseases, such as cancer [120], cardiovascular diseases [121] and neurological diseases [122]. Since the expression levels of circRNAs are usually associated with clinical and pathological features, these circRNAs have the potential to be biomarkers for diagnosis, prognosis and prediction [123-125]. In addition, the high stability of circRNAs allows them to be detected noninvasively in body fluids [126].

### 5.2 CircRNAs and Opioid Tolerance

circRNAs are highly expressed in the nervous system and are involved in many neurophysiological and pathological processes, such as neuronal development, learning, memory, and central nervous system-related diseases [127, 128]. Pain signal perception, transmission and feedback involve many aspects of the nervous system [129]. Several studies have reported that circRNAs play important roles in chronic pain. Pan *et al.* [130] found that circRNA-Filip11 interacts with miRNA-1224 to participate in the regulation of nociceptive sensation in inflammatory pain. Zhang *et al.* [131] found that circAnks1a contributed to central sensitivities and pain behaviors caused by nerve injury by regulating the expression of VEGFB in dorsal horn neurons at both the transcriptional and posttranscriptional levels. These studies suggest that circRNAs have novel roles in regulating the expression of genes associated with chronic pain. There have been no reports on whether circRNAs are involved in the development of morphine tolerance.

In addition to focusing on the role of miRNAs and lncRNAs in morphine tolerance, we also explored the function of circRNAs. Our previous study found a variety of circRNAs in the spinal cord to be significantly changed after morphine treatment: 2038 circRNAs were differentially expressed in morphine-tolerant rats, including 896 upregulated circRNAs and 1142 downregulated circRNAs, and it was confirmed that circRNA_005151, circRNA_010774, circRNA_014599, circRNA_012605 and circRNA_017999 were significantly downregulated and circRNA_008508 and circRNA_000047 were significantly upregulated in the spinal cord of morphine-tolerant rats [132]. Through bioinformatics analysis, we found that these differentially expressed circRNAs may be involved in the development of the nervous system and the transmission of neural signals and may also be related to glutamate synapses and MAPK signaling pathways. It is predicted that these circRNAs may form circRNA/microRNA/mRNA pathways; for example, they may inhibit the miR-181 family and indirectly regulate the TLR4 signaling pathway [132].

N6-methyladenosine (m6A), as a key posttranscriptional modification of RNA, can regulate the metabolism and functions of circRNAs. We explored the patterns of m6A methylation of circRNAs in the spinal cord of morphine-tolerant rats and found that the M6A modification of circRNAs may be involved in the pathogenesis of morphine tolerance [133]. These differentially expressed circRNAs may be novel potential targets for opioid tolerance. Although research in this area is still in the preliminary exploratory stage, as the first study to conduct circRNA differential expression detection and ceRNA analysis in morphine tolerance, our study provides a direction and clues for exploring the role of circRNAs in the development of morphine tolerance.

## CONCLUSION AND FUTURE PERSPECTIVES

**Fig. (1):**
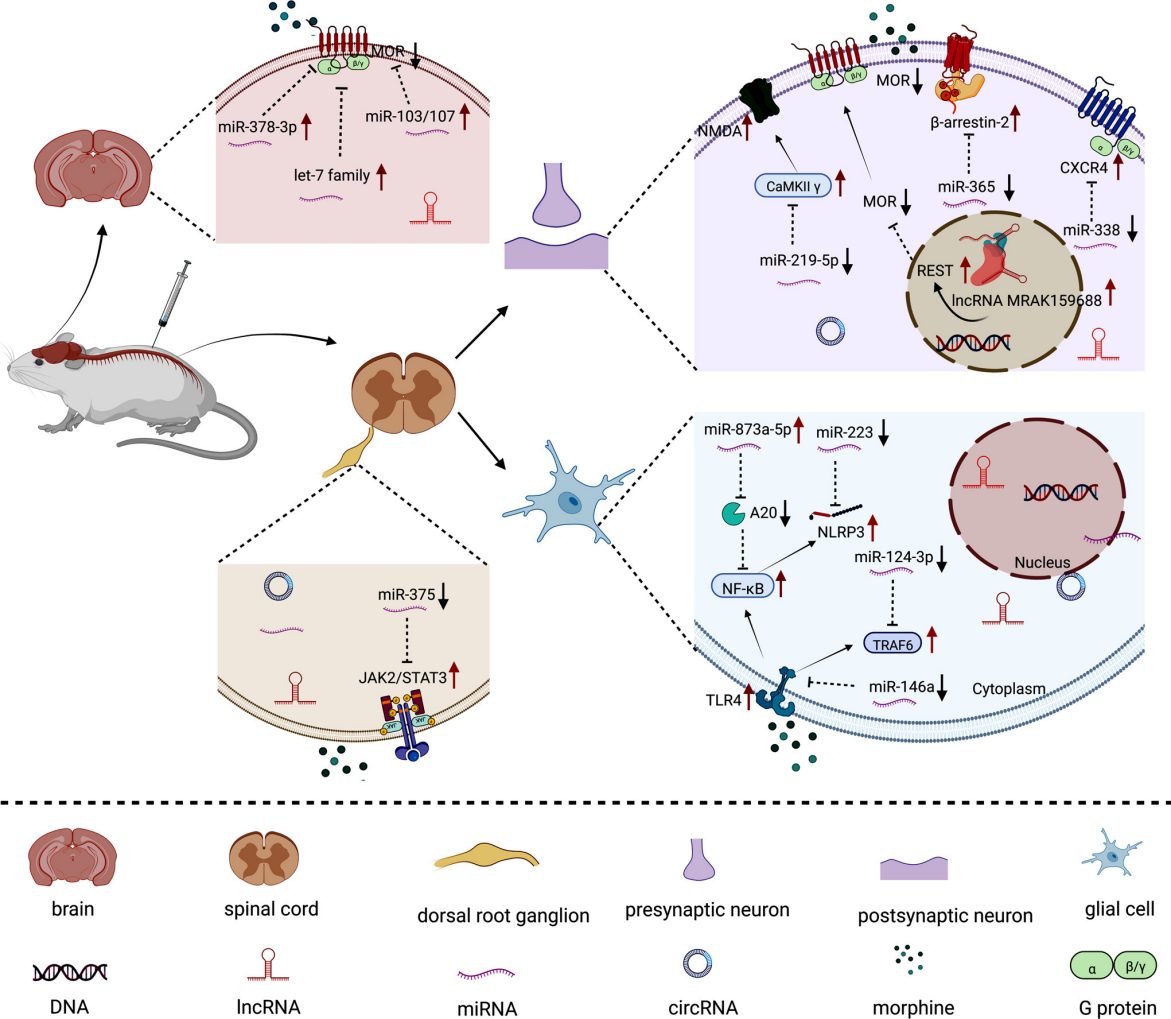
Schematic diagram of the possible mechanisms by which ncRNAs participate in opioid tolerance. After long-term administration of opioids, abnormally expressed miRNAs and lncRNAs in the brain, spinal cord and DRG of mice/rats promoted the development of morphine tolerance. The let-7 family, miR-103/107 and miR-378-3p were highly expressed in the brain, leading to the downregulation of MOR and morphine tolerance. The MORs in the spinal cord were phosphorylated, and miR-365 was significantly downregulated, leading to the upregulation of β-arrestin-2 and its binding to MORs, which led to MOR uncoupling from G protein, MOR desensitization and morphine tolerance. Downregulation of miR-219-5p led to the upregulation of CaMKII γ and promoted the expression and activation of NMDA, and downregulation of miR-338 promoted the upregulation of CXCR4, leading to morphine tolerance. The upregulated lncRNA MRAK159688 in the spinal cord participated in morphine tolerance by promoting REST-mediated MOR downregulation. miR-146a, miR-124-3p and miR-223 were significantly downregulated in the spinal cord, mediating the high expression of TLR4, TRAF6 and NLRP3 downstream, respectively, and participating in the development of morphine tolerance. miR-873a-5p was involved in morphine tolerance by inhibiting A20 expression. Morphine-induced downregulation of miR-375 in the DRG activated the JAK2/STAT3 pathway to promote morphine tolerance. MOR: mu opioid receptor; CaMKII γ: calmodulin-dependent protein kinase II γ; NMDA: N-methyl-d-aspartic acid receptor; CXCR4: CXC chemokine receptor 4; TLR4: toll-like receptor 4; TRAF6: tumor necrosis factor receptor-associated factor 6; NLRP3: NOD-like receptor protein 3; STAT3: signal transducer and activator of transcription 3.

Although the understanding of the molecular processes in which ncRNAs participate in cellular activities is incomplete, it is already clear that ncRNAs are widely involved in gene regulation, and they play important roles in a variety of physiological and pathological processes. Here, we have summarized the differential expression profiles and spatial dysregulations of biologically relevant ncRNAs following repeated injections of opioids (Fig. **1**), among which miRNAs are the most concerning, and their study is relatively mature. Although experimental animal studies of lncRNAs have just started, the role of circRNAs in opioid tolerance is still in the early stage of exploration, and the existing knowledge and findings will provide important clues and directions for further research. RNA mimics and anti-RNAs have shown promise in preclinical development. To determine the best RNA candidates or RNA targets for counteracting opioid tolerance, designing RNA delivery vectors, making therapeutic drugs that have higher stability, achieving tissue-specific targeting, and avoiding potential toxicity and off-target effects can contribute to the development of effective prevention approaches and the treatment of opioid tolerance in clinical practice.

Studies of the roles of ncRNAs in opioid tolerance have taken an important and meaningful first step. An important direction for future exploration of opioid tolerance is to identify more valuable noncoding transcripts. Functional validation of ncRNAs that have been identified and may be involved in the molecular mechanisms of opioid tolerance is necessary using preclinical models to assess the impact of the altered expression of ncRNAs more fully. Finally, potential functional noncoding RNA transcripts were selected as markers of a specific diagnosis or prognosis in patients with clinical opioid tolerance or as possible drug targets for clinical prevention and treatment of opioid tolerance.

## Figures and Tables

**Table 1:** Noncoding RNAs and opioid tolerance.

**Noncoding RNA**	**Opioid**	**Model**	**Site**	**Change**	**Target**	**References**
let-7 family	Morphine	Mouse	Brain	Increased	MOR	He *et al.* [49]
miR-103/107	Morphine	Mouse	Brain	Increased	MOR	Lu *et al.* [52]
miR-378-3p	Morphine	Mouse	Brain	Increased	MOR	Lu *et al.* [53]
miR-27a	Morphine	Mouse	Brain	Decreased	Serpini1	Tapocik *et al.* [54]
miR-146a	Morphine	Rat	Spinal cord	Decreased	TLR4	Wang *et al.* [62]
miR-124-3p	Dezocine	Rat	Spinal cord	Decreased	TRAF6	Huo *et al.* [65]
miR-223	Morphine	Rat	Spinal cord	Decreased	NLRP3	Xie *et al.* [70]
miR-365	Morphine	Rat	Spinal cord	Decreased	β-arrestin-2	Wang *et al.* [73]
miR-365	Morphine	Rat	Spinal cord	Decreased	ERK/CREB	Wu *et al.* [74]
miR-219-5p	Morphine	Rat	Spinal cord	Decreased	CaMKIIγ	Wang *et al.* [78]
miR-873a-5p	Morphine	Mouse	Spinal cord	Increased	A20	Huang *et al.* [80]
miR-338	Morphine	Rat	Spinal cord	Decreased	CXCR4	Mei *et al.* [83]
miR-375	Morphine	Mouse	DRG	Decreased	JAK2/STAT3	Li *et al.* [88]
LncRNA-MRAK159688	Morphine	Rat	Spinal cord	Increased	MOR	Deng *et al.* [110]

## LIST OF ABBREVIATIONS

AC: Adenylate Cyclase
cAMP: Cyclic Adenosine Monophosphate
circRNAs: Circular RNAs
DRG: Dorsal Root Ganglia
miRISC: miRNA-Induced Silencing Complex
miRNAs: microRNAs
